# Patterns and Drivers of Pollen Temperature Tolerance

**DOI:** 10.1111/pce.15207

**Published:** 2024-10-24

**Authors:** Donam Tushabe, Sergey Rosbakh

**Affiliations:** ^1^ Ecology and Conservation Biology, Institute of Plant Sciences University of Regensburg Regensburg Germany; ^2^ Department of Plant and Environmental Sciences, Faculty of Science University of Copenhagen Copenhagen Denmark

**Keywords:** climate change, cold, crop, heat, limits, pollen, reproduction, seed, temperature, tolerance

## Abstract

Pollen, a pivotal stage in the plant reproductive cycle, is highly sensitive to temperature fluctuations, impacting seed quality and quantity. While the importance of understanding pollen temperature limits (*Tmin*, *Topt*, *Tmax* – collectively PTLs) is recognized, a comprehensive synthesis of underlying drivers is lacking. Here, we examined PTLs, correlating them with vegetative tissue thermotolerance and assessing variability at the intra‐ and interspecific levels across 191 species with contrasting phylogeny, cultivation history, growth form and ecology. At the species level, the PTLs range from 9.0 to 42.4°C, with considerable differences among individual species. Vegetative tissue showed greater tolerance to both low and high temperatures than pollen. A significant, though weak, correlation was observed between PTLs and leaf temperature tolerance. Pollen heat tolerance was independent of that in leaves and stems. The greatest intraspecific variability was observed in pollen cold tolerance (*Tmin*), followed by *Topt* and *Tmax*. Phylogenetic analysis revealed family‐level conservation in all three pollen temperature tolerance measures. Climate emerged as a significant PTL driver of pollen cold tolerance, with species from colder and stable climates exhibiting enhanced cold tolerance. Cultivated and wild species did not differ in their pollen temperature tolerances. Herbaceous plants showed higher tolerance to high temperatures compared to shrubs and trees, potentially reflecting divergent thermal conditions during anthesis. This study provides the first formal analysis of complex relationships between pollen temperature limits, plant characteristics and environmental factors, providing crucial insights into climate change impacts on plant reproduction.

## Summary statement

The study examined pollen temperature tolerance in 191 plant species globally, finding a correlation between pollen and vegetative organ tolerance to low temperatures, phylogenetic conservation of pollen cold tolerance at the family level, and climate‐driven variations in pollen thermal limits.

## 1 Introduction

Globally, there is a growing concern in the scientific community about the adverse impacts of climate change on the reproduction processes of plants (Hedhly, Hormaza, and Herrero 2009; Fahad et al. 2017; Piao et al. 2019). Climate change‐driven extremes in temperature, such as cold spells and heatwaves occurring during critical plant developmental stages related to seed production, consistently lead to diminished seed quality and quantity (Neuner et al. 2013; Hatfield and Prueger 2015; Raza et al. 2019; Yadav et al. 2022). Consequently, the altered rates of viable seed production are anticipated to exert profound ecological impacts on plant population dynamics, with potential implications for species demography and long‐term survival. For instance, in cases where population growth hinges on seed availability, persistently low seed production may lead to a decline in species abundance and even the eventual extinction of certain plant species (Turnbull, Crawley, and Rees 2000; Willis et al. 2008). Moreover, fluctuations in fruit and seed production in wild plants can ripple through ecosystems, impacting various trophic levels and resulting in intricate interactions with numerous animal species. Reduced plant reproduction can affect birds and insects that rely on seeds and fruits as food sources, with downstream consequences for mammals and the prevalence of human pathogens (Lewis et al. 2014; Bogdziewicz, Zwolak, and Crone 2016). Lastly, climate‐induced alterations in the reproductive performance of cultivated plants may have detrimental implications for food security, potentially resulting in significant reductions in crop yields in the coming years (Ray et al. 2019; Caparas et al. 2021).

While temperature exerts control over all aspects of seed production (Slafer et al. 2015), pollen, the male gametophyte, emerges as the most temperature‐sensitive within the plant reproductive cycle when compared to other tissues and developmental stages (Sharkey and Schrader 2006; Hedhly 2011; Prasad, Bheemanahalli, and Jagadish 2017). Its susceptibility varies across its growth stages, with early phases of division being the most sensitive and mature pollen the most resilient (Zinn, Tunc‐Ozdemir, and Harper 2010; Chaturvedi et al. 2021). The heightened pollen sensitivity, as opposed to ovules, is attributed to several factors, including its comparatively small size, haploid set of chromosomes, lack of protective tissue and direct exposure to the environment (Bedinger 1992; Pacini and Dolferus 2016). Consequently, an array of experimental studies has demonstrated that even mild temperature stress applied at various stages of pollen development (anther wall development, microsporogenesis, microgametogenesis, pollen germination [PG] and pollen tube growth [PTG]) results in a substantial decline in pollen performance, often yielding irreversible effects (Kakani et al. 2002; Sato, Peet, and Thomas 2002; Raja et al. 2019; Tushabe et al. 2023). These findings collectively underscore that pollen sensitivity to both low and high temperatures (‘cold and heat tolerance’) is a pivotal limiting factor in seed productivity and is particularly susceptible to the effects of global climate change (Hedhly, Hormaza, and Herrero 2009; Hassan et al. 2021; Jagadish, Way, and Sharkey 2021).

Understanding pollen temperature tolerance is a subject of considerable interest among plant scientists spanning various disciplines (Kakani et al. 2005; Mesihovic et al. 2016; Rosbakh et al. 2018; Djanaguiraman et al. 2019). This interest is particularly pronounced in the context of ongoing climate change, as it holds the potential to shape the future of both wild plant conservation and agricultural productivity. However, despite the pivotal role of this trait in plant reproductive processes, our knowledge regarding the critical temperature thresholds of pollen remains relatively limited. Equally elusive is our understanding of the extent of variability in these thresholds and the underlying factors contributing to such variability. The existing body of literature on pollen thermal limits predominantly concentrates on either a select few cultivars of a single crop species (e.g., Kakani et al. 2002; Coast et al. 2016; Paupière et al. 2017) or a limited assortment of wild species confined to specific environments (e.g., Rosbakh and Poschlod 2016; Wagner et al. 2016). This focused approach, although valuable in its own regard, presents challenges for researchers seeking to draw any general conclusions on the adaptability and susceptibility of pollen temperature tolerances to changing environmental conditions, both in space and time.

Here we bridge this knowledge gap by examining patterns and drivers of pollen cold and heat tolerance across multiple populations and species of wild and cultivated plants occurring worldwide. To achieve this, we harness a data set encompassing crucial temperature parameters for PG and PGT – including minimum, optimal and maximum temperatures (cardinal temperatures) – for 191 species measured in 636 populations and/or cultivars, along with > 500 000 georeferenced species occurrences across main world's biomes. Our first objective is to investigate the temperature limits governing pollen performance and explore their potential correlations with the thermotolerance observed in vegetative tissues (*Aim 1*). While it has been often suggested that all stages of plant sexual regeneration function within a narrower range compared to leaves, stems and roots (e.g., Luo 2011; Nievola et al. 2017), this assumption has been poorly empirically examined, with only a few examples like Neuner and Buchner (2012).

Next, we explore the presence and extent of intraspecific variability in pollen temperature tolerance (*Aim 2*). Previous research has shown that plants tend to adapt their pollen performance to the local growing conditions, with example of populations from colder regions exhibiting better pollen cold tolerance as opposed to their counterparts from warmer habitats (Morrison et al. 2016; Ranasinghe, Kumarathunge, and Kiriwandeniya 2018; Zebro, Kang, and Heo 2023). However, these investigations have typically centred on individual species or focused solely on specific temperature thresholds, such as minimum or maximum temperatures. Consequently, we are still lacking a comprehensive understanding of the adaptability and plasticity of pollen performance in response to changing thermal conditions, both temporally and spatially.

In the next part of the analysis, we tackled drivers of pollen thermal limits at the species (interspecific) level. First, we tested for the presence of a phylogenetic signal in the pollen temperature tolerance data (*Aim 3*). The rationale for this analysis stems from the well‐established understanding that closely related taxa often retain ecological traits and environmental preferences of their ancestors (Crisp et al. 2009; Burns and Strauss 2011; Kamilar and Cooper 2013; Liu et al. 2015). Accordingly, we anticipated that pollen thermal limits would exhibit a certain degree of phylogenetic conservation. Subsequently, we assessed whether interspecific variation in pollen tolerance to both low and high temperatures could be attributed to specific temperature extremes encountered in the locations where these plants grow (*Aim 4*). Building upon insights from prior research (Rosbakh and Poschlod 2016; Zhu et al. 2018; Lancaster and Humphreys 2020; Sentinella et al. 2020), we posited that pollen, inhabiting climates characterized by generally low temperatures and/or substantial temperature fluctuations might have evolved enhanced temperature tolerance mechanisms. These adaptations would enable them to cope with short‐ and long‐term temperature shifts, including frost events, heatwaves and seasonal temperature variations. We further considered differences in pollen thermal limits for wild and cultivated plants, as the adaptability to local climate conditions can differ due to natural selection pressures for wild plants and selective breeding in cultivated varieties (Lippmann et al. 2019). Also, we tested for differences in pollen thermotolerance in plants with different growth forms, as relatively short herbs tend to experience warmer thermal environments compared to tall trees. This difference arises from the ground‐level radiative heating experienced by herbs in the near‐surface air, in contrast to tall trees, which have a thermal coupling with the ambient atmosphere (Geiger, Aron, and Todhunter 2009; Treml, Hejda, and Kašpar 2019).

## 2 Materials and Methods

### 2.1 Data Collection

**Figure 1:**
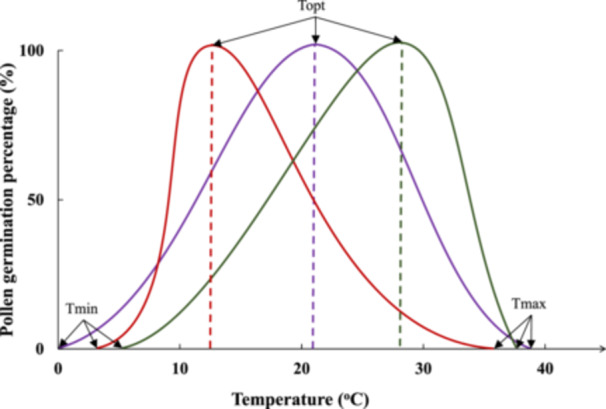
Potential pollen responses to cultivation temperature gradient in three species, shown by different coloured curves (purple, red, green). *Tmin*, *Topt* and *Tmax* denote the species‐specific temperatures (‘cardinal temperatures’), at which pollen germination and pollen tube growth is initiated (*Tmin*), terminated (*Tmax*) and has its highest proportion and growth rate (*Topt*), respectively.

In this study, we define pollen tolerance to low and high temperatures as temperatures falling below or exceeding, respectively, that cause stress, affecting pollen morphological, physiological, biochemical and molecular properties, and ultimately its performance (Wahid et al. 2007; Bewley and Black 2013; Hasanuzzaman et al. 2013; Liu et al. 2023). Moreover, we investigate the optimal temperature range, wherein pollen exhibits its peak performance. This ideal range corresponds to the conditions fostering the highest proportion of germinated pollen grains and the fastest PTG. Pollen temperature tolerances are frequently assessed through three key temperatures, which are *Tmin* (the minimum), *Topt* (the optimum) and *Tmax* (the maximum). These parameters are essential for understanding PG and PTG, as highlighted in studies such as Kakani et al. (2005) and Rosbakh and Poschlod (2016). *Tmin* and *Tmax* represent the temperature extremes at which neither pollen grains can germinate, nor pollen tubes can grow. In contrast, *Topt* is the temperature range wherein a species' pollen grains exhibit their highest germination rates, and PTG is maximized in terms of length (Figure 1).

To compile the data set on pollen thermotolerance, we first extracted available information on cardinal temperatures for PG and PTG by reviewing all studies published from 1933 to 2020 via search in the Web of Science database with the keywords ‘pollen’, ‘germination’ and ‘temperature’. The search resulted in a total of 1268 studies, out of which 91 contained information on the PG and PTG cardinal temperatures that were subsequently extracted. From each selected publication, we extracted data on study species and their cultivars (if applicable), the cardinal temperatures for PG and/or PTG, whether pollen cultivation was conducted in vivo (on the stigma of flowers within living plants) or in vitro (on a germination medium in the laboratory), the range of test temperatures (e.g., 0–40 or 20–35°C), and the statistical modelling technique employed to derive temperature threshold estimates (e.g., bilinear, beta or quadratic models). In cases where the temperatures were given as a range, the average values of these ranges were used. Importantly, differences in study methods, including pollen cultivation (in vivo vs. in vitro), temperature range tested, and statistical models used, can affect temperature threshold estimates. In vivo cultivation on living plants captures natural stress responses, while in vitro cultivation in controlled environments may not fully reflect natural conditions, leading to different threshold estimates. Additionally, studies with broader temperature ranges might capture more extreme responses, affecting thresholds compared to those with narrower ranges. Different statistical models can fit the data differently, further influencing threshold estimates.

To enhance the data set, we integrated additional experimental data concerning the cardinal temperatures for PTG from a set of 91 Central European plant species collected at the University of Regensburg, Germany, in 2014–2020 (S. Rosbakh, unpublished); the detailed information on pollen cultivation is given in Rosbakh and Poschlod (2016). Briefly, pollen samples were hydrated using a saturated KCl solution to prevent bursting, then mixed with germination media varying in sucrose concentrations (Tushabe and Rosbakh 2021). In vitro PG experiments were conducted at nine different temperatures (5–34°C) using a thermogradient table. After 18 h, germination was terminated, and the percentage of PG and PTG was determined by examining pollen grains per four replicates. Germination was defined as a pollen tube length at least double the grain diameter. A generalized plant growth model was then fitted to the data to determine the minimum (*Tmin*), optimum (*Topt*) and maximum (*Tmax*) temperatures for PTG.

The species taxonomy was harmonized against the Plant List (The Plant List 2013) with the R package *taxonstand* (Cayuela et al. 2021). In total, the consolidated data set encompasses 636 entries for 191 species from 128 genera and 57 distinct families. Due to the strong and statistically significant correlations observed between the cardinal temperatures for PG and PTG at both the intra‐ and interspecific levels (Appendix S1: Figure A1), we used the respective cardinal temperatures of both PG and PTG – *Tmin*, *Topt* and *Tmax* – to represent overall pollen performance. To test for phylogenetic signal (*Aim 3*), we used arithmetic mean values aggregated at the species level.

Each species was then characterized in terms of the thermal conditions that plants, and therefore pollen, experience in their habitats, cultivation status (wild or cultivated) and growth form (herbaceous vs. woody). To characterize climatic requirements for each species, we used the geographic coordinates from the Global Biodiversity Information Facility (GBIF) using the package *rgbif* (Chamberlain et al. 2023) in R software v.4.3.0 (R Core Team 2023). Only species with georeferenced locations obtained from known herbarium vouchers were considered in the analysis. For each coordinate, we extracted data on mean annual temperature (MAT; ‘BIO1’) and temperature annual range (TAR; ‘BIO7’) from the WorldClim database (Fick and Hijmans 2017). MAT represents overall thermal conditions, with lower values indicating colder habitats at higher latitudes and elevations, while TAR reflects climate seasonality, with higher values indicating more continental climates with pronounced cold seasons. Although direct temperature measurements during the flowering period would most accurately capture the environmental conditions affecting pollen thermotolerance, such data were unavailable for most observed locations due to the variability in flowering phenology across large biogeographic gradients. Thus, MAT and TAR served as proxies, allowing us to analyze a broad range of habitats and maintain a wide geographic scope in our study. Species macroclimate temperature preferences were then expressed as an average MAT and TAR and as a median overall species occurrence in our data set (i.e., climatic envelopes).

To accomplish our first objective of comparing temperature tolerance between pollen grains and vegetative organs, we conducted an additional search to identify studies reporting the lethal *Tmin* and *Tmax* thermal limits for leaves, stems and whole plants of species for which we had available pollen temperature tolerance data. Optimal temperatures (*Topt*) for vegetative tissues have been rarely studied and thus were omitted from the analysis. This targeted search was executed in the Web of Science database, utilizing keywords ‘vegetative’, ‘thermotolerance’ and the corresponding species name. This search yielded a data set consisting of 13 species with *Tmin* thermal limits and 30 species with *Tmax* thermal limits for vegetative organs (almost exclusively leaves), which were concurrently represented in our pollen temperature tolerance data set.

### 2.2 Data Analysis

All statistical analyses were performed using R software version 4.3.0 (R Core Team 2023).

### 2.3 Relationship Between the Pollen and Vegetative Organs' Temperature Tolerance (Aim 1)

First, we tested whether temperature tolerance to low and high temperatures is different in pollen and vegetative organs with the help of a linear mixed‐effect model with family included as a random effect to account for potential phylogenetic autocorrelation (i.e., Temperature ~ Organ + (1|Family)). After that, we examined the relationship between temperature tolerance in pollen and vegetative organs, by fitting a linear mixed‐effect model with family included as a random effect.

### 2.4 Intraspecific Pollen Temperature Tolerance Variability (Aim 2)

To explore intraspecific variability in pollen temperature tolerance, we focused on 11 species from our data set that had data available for more than 20 populations. Importantly, all these species were cultivated (Appendix S2: Table A1; most of the wild species in the data set were represented by only a single study population), and these populations effectively correspond to cultivars. To visualize the pollen thermal limits variability within the selected species, we used boxplots integrated into the violin plot. Additionally, to estimate the degree of intraspecific variation in pollen *Tmin*, *Topt* and *Tmax*, we calculated coefficient of variation (CV) for each temperature for each of the species. A linear model in combination with post hoc Tukey test was used to test for the differences in CVs among the pollen temperature tolerance. The CVs values were log‐transformed, to improve the normality of residuals; all model requirements were met.

### 2.5 Phylogenetic Signal in Pollen Temperature Tolerance (Aim 3)

Pollen temperature tolerance data were then plotted on the species' phylogeny using the package *phytools* (Revell 2012). The phylogenetic tree for the study species was compiled using the package *V.PhyloMaker* (Jin and Qian 2019).

To test whether the pollen thermal limits were phylogenetically constrained, we first calculated Blomberg's *K*‐statistics, Brownian motion‐based metric of the strength of the phylogenetic signal (Blomberg, Garland, and Ives 2003), using the phylosignal function in the *picante* library (Kembel et al. 2010). K = 1 indicates that closely related species have trait values that are similar to those expected given Brownian motion; *K* < 1 indicates that closely related species have trait values that are less similar than expected given a Brownian model of evolution. Additionally, we run Moran's *I* test for *Tmin, Topt and Tmax*, an alternative estimate of a phylogenetic signal indicating how phylogenetic signature changes across the phylogeny (Gittleman and Kot 1990). The resulting values of this analysis do not offer any quantitative interpretation of the phylogenetic signal, because the expected value of the statistic under the assumed model is unknown a priori. However, stronger deviations from zero indicate stronger relationships between trait values and the phylogeny (Münkemüller et al. 2012). The phylogenetic autocorrelation in the data was estimated at three taxonomic levels: family, class and order.

### 2.6 Interspecific Trait Variation (Aim 4)

To estimate the variability in pollen temperature tolerance variability at the species level, we fitted three linear mixed effects models with one of the temperatures (*Tmin*, *Topt* or *Tmax*) being the response variable in the corresponding models. The model predictors (i.e., fixed effects) were MAT and TAR, cultivation status and growth form. To account for the phylogenetic signal (see above), the family was included in all models as a random factor. Species and source of pollen cardinal temperatures (i.e., study) were included as random factors, to account for both among and within study variance. That is, the model syntax was Pollen thermotolerance ~MAT + TAR + Cultivation status + Woodiness + (1|Family) + (1|Species) + (1|Study). The models were fitted using the packages *lme4* (Bates et al. 2015), *lmertest* (Kuznetsova, Brockhoff, and Christensen 2017) and *mumin* (Bartoń 2023). Differences in pollen temperature tolerances among plant groups with different characteristics (e.g., cultivation status and growth form) were estimated with the help of the post hoc Tukey test (*p* < 0.05), implemented in the packages *emmeans* and *multcomp* (Hothorn, Bretz, and Westfall 2008; Lenth 2023). All numeric variables were centred and scaled to unit variance so their effects could be compared.

## 3 Results

### 3.1 Temperature Limits of Pollen Temperature Tolerance

Of the 636 entries in the data set, representing 191 species from 128 genera and 57 families, the most common family was Fabaceae (22%) and *Glycine max* (L.) Merr. the most studied species (14%). In general, studies on cultivated species (78%) were more frequent than those on wild species (22%).

**Figure 2:**
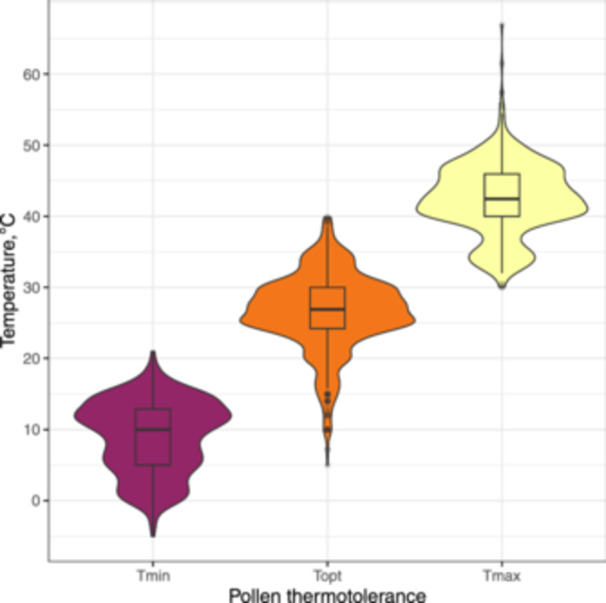
Average pollen thermal limits for 191 species in the data set. *Tmin*, *Topt* and *Tmax* are minimal, optimal and maximal temperatures of pollen performance. Violin plots show data distribution, while box plots focus on summary statistics and outliers. [Color figure can be viewed at wileyonlinelibrary.com]

The pollen cold tolerance (*Tmin*) ranged from −5°C in early‐flowering dwarf shrub *Polygala chamaebuxus* occurring on calcareous soils in temperate climate to 21°C in *Juglans nigra*, a tree grown and cultivated in temperature climate of North America, with an average of 9.0°C. The 5th and 95th percentiles of the *Tmin* values distribution ranged from 0 to 15.8°C (Figure 2).

On average, the optimal temperature of pollen performance (*Topt*) was 26.5°C, ranging from 5.0°C in *Prunus armeniaca* (apricot; a fruit tree cultivated in continental climates) to 39.9°C in *G. max* (soybean) grown in various climates. The 5th and 95th percentiles of the *Topt* values distribution ranged from 16.1 to 34.8°C (Figure 2).

The limit of pollen heat tolerance ranged from 30.0°C in *Betula pendula*, a wild tree common in temperate and boreal climates with early flowering phenology, to 66.9°C in *Saintpaulia ionantha* (African violet), an ornamental plant native to eastern tropical Africa. The 5th and 95th percentiles of the *Tmax* values distribution ranged from 33.5 to 49.6°C (Figure 2). Averaged over all species, the *Tmax* value was 42.4°C.

### 3.2 Relationship Between the Pollen and Vegetative Organs' Temperature Tolerance (Aim 1)

**Figure 3:**
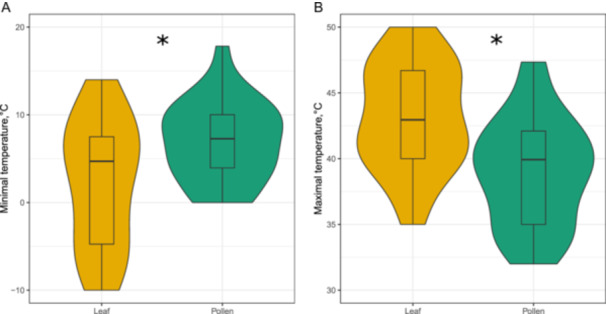
Comparison of pollen and vegetative organ tolerance to (A) minimal (*Tmin*, *n* = 13) and (B) maximal temperatures (*Tmax* = 30). Violin plots show data distribution, while box plots focus on summary statistics and outliers. Asterisks indicate statistically significant differences as inferred from linear mixed‐effect models. [Color figure can be viewed at wileyonlinelibrary.com]

The linear models revealed that pollen and vegetative organ tolerance to low temperatures and high temperatures differed significantly (Figure 3). On average, pollen *Tmin* values were 11.6 ± 2.3°C, whereas those of vegetative organs were 5.5 ± 2.4°C (Figure 3A). Vegetative organs were also found to better tolerate high temperatures as compared to pollen (*Tmax* values of 46.1 ± 1.2°C and 38.9 ± 1.2°C, respectively; Figure 3B).

**Figure 4:**
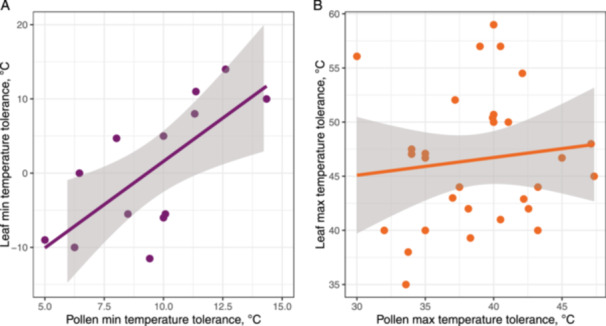
Correlation between (A) minimal (*Tmin*, *n* = 13) and (B) maximal (*Tmax*, *n* = 30) pollen and vegetative organ temperature tolerance. Shaded areas denote 95% confidence intervals. [Color figure can be viewed at wileyonlinelibrary.com]

We also found that pollen and vegetative organ tolerance to low temperatures were significantly, positively but weakly correlated with each other (Figure 4A, *r* ^2^ = 0.14, *p* = 0.023, *n* = 13). Contrastingly, we did not detect any statistically significant differences in high‐temperature tolerances between vegetative organs and pollen (Figure 4B, *r* ^2^ = 0.02, *p* = 0.45, *n* = 30).

### 3.3 Intraspecific Variability in Pollen Temperature Tolerance (Aim 2)

**Figure 5:**
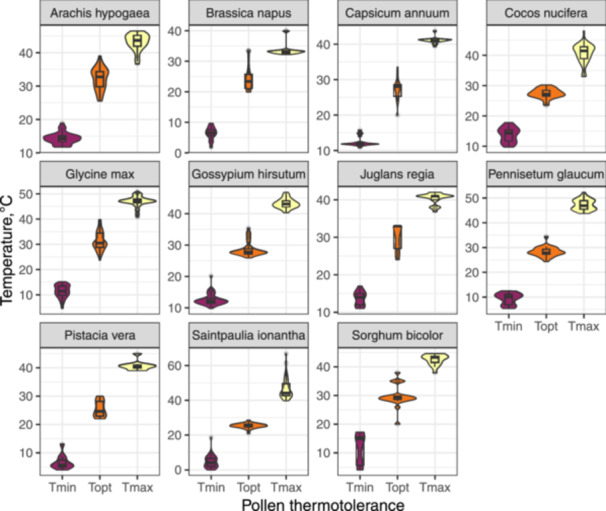
Intraspecific variation in pollen tolerance to low and high temperatures in 11 cultivated plant species. Violin plots show data distribution, while box plots focus on summary statistics and outliers. [Color figure can be viewed at wileyonlinelibrary.com]

Eleven species with data for more than 20 cultivars were tested for intraspecific variability in pollen thermal limits (Figure 5; Appendix S2: Table A1). Averaged over all species and temperatures, CV ranged from 1.73 (*Tmax* in *Capsicum annuum*) to 72.8 (*Tmin* in *S. ionantha*). The linear model revealed that the degree of intraspecific variability, as deduced from the CVs, was significantly different (*p* < 0.01) among all three thermotolerance limits, being the largest in *Tmin* (3.1) followed by *Topt* (2.2) and *Tmax* (1.5).

### 3.4 Phylogenetic Signal in Pollen Temperature Tolerance (Aim 3)

The distribution of pollen thermal limits across the phylogenetic tree is shown in Appendix S1: Figure A2. Blomberg's K indicated a weak but significant phylogenetic signal in all three temperatures (*Tmin*: *K* = 0.13, *p* = 0.001, *n* = 147; *Topt*: *K* = 0.11, *p* = 0.036, *n* = 175; *Tmax*: *K* = 0.14, *p* = 0.005, *n* = 139). The Moran's *I* test revealed that the plants from the same family tended to share similar pollen thermotolerance (*Tmin*: Moran's *I* = 0.3, *p* < 0.001; *Topt*: *I* = 0.30, *p* < 0.001; *Tmax*: *I* = 0.19, *p* = 0.006), whereas these values were randomly distributed across orders and classes.

### 3.5 Interspecific Trait Variability (Aim 4)

**Figure 6:**
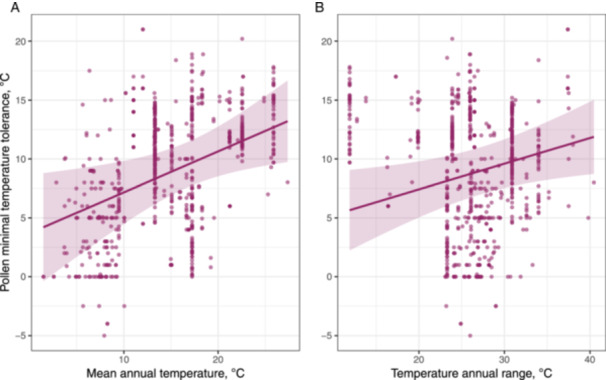
Variation of pollen cold tolerance (*Tmin*) along gradients mean annual temperature (A) and temperature annual range (B) as deduced from linear mixed‐effects models. Shaded areas denote 95% confidence interval. [Color figure can be viewed at wileyonlinelibrary.com]

The linear mixed‐effects models revealed that habitat temperature conditions expressed as MAT and TAR had statistically significant effects only on minimal (*Tmin*) temperatures of pollen performance (Table 1; Figure 6). Specifically, plants occurring in colder and stable climates tended to have smaller *Tmin* values as compared to their counterparts from warmer and more seasonal climates. Species with different cultivation statuses did not differ in their cardinal temperatures. Herbaceous species were found to have significantly larger *Tmax* values as compared to trees and shrubs (39.4 and 37.3°C, respectively).

**Table 1:** Effects of mean annual temperature, temperature annual range, plant cultivation status and growth form on pollen thermal limits of the 191 species as determined from generalized linear mixed‐effect models.

Predictors	Pollen cold tolerance (*Tmin*)			Optimal temperature of pollen performance (*Topt*)			Pollen heat tolerance (*Tmax*)		
	Estimates	CI	p	Estimates	CI	p	Estimates	CI	p
Intercept	8.80	6.80–10.80	**< 0.001**	25.12	23.58–26.67	**< 0.001**	41.41	39.93–42.89	**< 0.001**
Mean annual temperature (BIO1)	1.97	0.43–3.52	**0.012**	0.82	−0.41–2.05	0.19	0.68	−0.41–1.76	0.22
Temperature annual range (BIO7)	1.17	0.19–2.15	**0.019**	0.16	−0.71–1.04	0.711	0.65	−0.03–1.33	0.06
Cultivation status	−2.12	−6.24–1.99	0.311	−1.66	−5.05–1.72	0.336	−4.00	−8.23–0.23	0.064
Growth form	0.65	−1.07–2.37	0.457	0.69	−0.89–2.26	0.391	−2.13	−3.41– −0.85	**0.001**
Marginal R^2^	0.17			0.05			0.20		
Conditional R^2^	0.88			0.75			0.72		

*Note:* Bold values indicate significant treatment effects (*p* < 0.05). CI–95% confidence intervals. Marginal *R* ^2^–the variance explained by the model fixed factors only (listed in the first column); conditional *R* ^2^–the variances explained by the full model (i.e., the listed fixed factors plus phylogeny (family), species (e.g., uncaptured differences in species biology and ecology) and study, from which data on pollen cardinal temperatures were extracted.

## 4 Discussion

Traditionally, research on the temperature limits of plant performance has mainly focused on vegetative and/or experimentally accessible organs, such as leaves (Neuner and Buchner 2012; Geange et al. 2021), stems (Sakai and Larcher 2012) or seeds (Sentinella et al. 2020) rather than on gametophytes (Rosbakh et al. 2018). While there is a substantial body of experimental work addressing pollen response to temperature stress (e.g., Raja et al. 2019; Chaturvedi et al. 2021; Tushabe et al. 2023; Zebro, Kang, and Heo 2023), these findings have seldom been consolidated and systematically analyzed across various scales. Our study bridges this gap by presenting the first comprehensive assessment of thermal limits of pollen performance and their drivers across multiple populations and species of wild and cultivated plants occurring worldwide.

### 4.1 Temperature Limits of Pollen Performance

Our analysis reveals that, generally, pollen performance is limited to temperatures ranging from 9.0°C (*Tmin*) to 42.4°C (*Tmax*), with 26.5°C being the optimal temperature for PG and PTG (*Topt*). Also, several plant species in our data set have seemingly evolved pollen that can tolerate relatively low (the lowest *Tmin* value of −5°C) and high (the highest *Tmax* value of 66.9°C) temperatures. These data, combined with our formal comparison of pollen and vegetative tissue tolerances in a smaller data set (Figure 3), suggest that, on average, pollen has a narrower temperature tolerance range, as leaves were found to tolerate better both lower and higher temperatures. In general, plants can maintain their growth and development over a wide range of temperatures, approximately between −10 and +45°C (Larcher 2003; Luo 2011; Nievola et al. 2017; Lancaster and Humphreys 2020), while tissues of species growing in the most extreme biomes can survival temperatures between −60°C and +60°C for short durations (Larcher 2003; Nievola et al. 2017; Geange et al. 2021). We attribute the narrower pollen temperature tolerance to the lack of protective tissue in pollen grains, their comparatively small size and short lifespan, haploid set of chromosomes, and general sensitivity of cells to high‐temperature stress (Bedinger 1992; Dafni and Firmage 2000; Araújo et al. 2013; Lohani et al. 2021). Consequently, these features explain why most of the *Tmin* values are above 0°C (germinated pollen grain and growing pollen tubes have limited opportunities to repair frost damage; Wagner et al. 2016) and below 40°C (adaptive changes in lipid composition of membranes and production of heat shock proteins are impaired at temperatures around and above 45°C; Araújo et al. 2013). Thus, to circumvent the detrimental impacts of cold and heat stress on pollen germination and growth, plants coordinate the timing of anthesis with ambient temperature conditions (e.g., Riihimäki and Savolainen 2004), both seasonal and diurnal, which are optimal for pollen performance. In essence, pollen adaptation to extreme temperatures is unnecessary since anthesis does not occur during freezing winters or scorching summers.

### 4.2 Pollen and Leaves Do Not Share Similar Tolerance to Low and High Temperatures

Analyzing the potential correlation between temperature tolerance in vegetative tissues and pollen in the subset of species (*n* = 13 for *Tmin* and *n* = 30 for *Tmax*; Figure 4), we see that plants with leaves adapted to extremely low temperatures also tend to produce pollen grains with comparable temperature tolerance. Despite their distinct roles in the life cycle of plants, leaves and pollen share several characteristics at the molecular level that could contribute to their respective adaptive responses to low temperatures. Such key molecular similarities reflect their common evolutionary heritage and shared biological processes that include antioxidant defence systems to regulate the levels of reactive oxygen species (Gill and Tuteja 2010; Das and Roychoudhury 2014), synthesis of protective proteins (e.g., antifreeze proteins; Lee and Lee 2003) and adjustments in cell membrane lipid composition (Narayanan, Prasad, and Welti 2018). However, the relatively weak correlation between pollen and leaf tolerance to low temperatures (*R*² = 0.14) suggests that these molecular mechanisms differ in their extent and efficiency due to the divergent roles of pollen, which is specialized for reproduction with a short lifespan, and leaves, which support long‐term plant growth.

The lack of a significant relationship between the leaf and pollen heat tolerance suggests that these two tissues might employ different mechanisms to reduce the negative effects of high‐temperature exposure. Particularly, vegetative organs as complex, multicellular and interconnected formations have many more opportunities to respond to heat stress by modifying their anatomy (e.g., presence of trichomes reducing heat absorption), morphology (e.g., reduced leaf size and altered leaf orientation) and physiology (e.g., stomatal regulation and more efficient transport of water and metabolites among the organs) as compared to simply organized single‐celled pollen grains. Additionally, leaves potentially possess a greater capacity for rapid acclimation due to enduring repeated diurnal cycles throughout their lifespan, in contrast to pollen grains, which germinate and develop over a shorter period, necessitating less acclimation. As a result, pollen may exhibit less variability in thermal tolerance compared to leaves, potentially making correlations between them difficult, especially if the pollen and leaves have not experienced similar temperature histories. Thus, it remains unclear whether plant vegetative heat‐tolerance can reliably predict pollen thermotolerance, a key trait in, for example, biogeography (Rosbakh and Poschlod 2016; Rosbakh et al. 2018) or plant breeding (Kakani et al. 2002, 2005).

### 4.3 Intraspecific Variability in Pollen Temperature Tolerance

The assessment of intraspecific variability in pollen cardinal temperatures within the subset of 11 cultivated species revealed a notable degree of plasticity in pollen temperature tolerance. Although we were not able to ascertain the specific climate conditions under which these cultivars were grown, we speculate that this variability primarily stems from adaptations to local growing conditions, facilitated by an active breeding process towards better tolerance to temperature extremes (e.g., Kakani et al. 2002, 2005). It is plausible that cultivars developed for and cultivated in colder and warmer regions are more likely to display enhanced tolerance to low and high temperatures, respectively (Gajanayake et al. 2011; Morrison et al. 2016; Ranasinghe, Kumarathunge, and Kiriwandeniya 2018). The question of whether the observed intraspecific variability in pollen temperature tolerance is primarily driven by genetic differences, phenotypic plasticity, or a combination of both, remains unanswered due to the lack of corresponding research. Future investigations, such as plant cultivation in controlled environments or common gardens, combined with population genetic studies, are needed to shed light on this complex issue.

One of the most interesting findings of the study was the significantly larger variation in pollen cold tolerances across different species populations, while *Topt* and *Tmax* values were more stable. This observation aligns with previous research on intraspecific pollen performance of individual species (Kakani et al. 2005; Salem et al. 2007; Singh et al. 2008) and broader studies on whole‐plant temperature tolerance spanning multiple species (Araújo et al. 2013; Lancaster and Humphreys 2020; Bennett et al. 2021; Geange et al. 2021). Specifically, the consistency of *Tmax* values across the study populations supports the theory that plant heat tolerance is constrained by the destabilizing impact of temperatures surpassing 45°C on cell membranes and proteins, whereas plants demonstrate greater flexibility in their adaptive response to low temperatures (Araújo et al. 2013; Bennett et al. 2021). Additionally, the asymmetry in pollen tolerance to low and high temperatures may be augmented by different selective pressures on thermal limits; maximum habitat temperatures tend to exhibit less variation across contemporary biomes compared to minimum temperatures (Bennett et al. 2021). Finally, the spread of agriculture from warmer to colder climates has exerted additional selection pressure on pollen cold tolerance. The increased variability of pollen cold tolerance might reflect the crop breeding efforts at high latitudes and elevations aimed at adapting the studied species to novel, colder climates (Kakani et al. 2002, 2005). Irrespective of the underlying cause (plasticity or innate genetic variation among genotypes), the relatively lower variation of pollen heat tolerance raises concerns about the limited adaptive potential of plant sexual regeneration, particularly the male gametophyte performance, to the increasing high‐temperature stress resulting from recent climate warming. In the long term, the impaired fruit and seed production could have adverse effects on plant population dynamics (Turnbull, Crawley, and Rees 2000), plant–granivore interactions (Lewis et al. 2014; Bogdziewicz, Zwolak, and Crone 2016), and ultimately, the food security for human populations (Seppelt et al. 2022).

### 4.4 Phylogenetic Patterns in Pollen Thermal Limits

Gaining insights into whether and to what extent pollen thermal limits are conserved across broad taxonomic groups can enhance our ability to predict how plant sexual regeneration might be impacted by current and future climates. Often, species preserve the ecological traits and environmental distributions inherited from their ancestors, with the tendency for closely related species to share similar values for a given trait than distantly related species (Crisp et al. 2009; Burns and Strauss 2011; Liu et al. 2015). Analyzing the distribution of pollen cardinal temperatures across the phylogenetic tree, we revealed that all three cardinal temperatures – *Tmin*, *Topt* and *Tmax* – showed some degree of phylogenetic conservation at the family level. The trend in pollen low‐temperature tolerance supports the ‘deep‐time climate legacies’ hypothesis, which suggests that species whose ancestors originated in colder paleoclimates tend to exhibit better tolerance to colder temperatures compared to species with warm thermal ancestry (Bennett et al. 2021). However, the phylogenetic conservatism of *Tmax* values contradicts another tenet of the ‘deep‐time climate legacies’ hypothesis, suggesting that physiological constraints (e.g., cell membrane protein functioning at temperatures above 45°C; see above) restrict the evolution of heat tolerance in living organisms (Hamilton 1973). In this context, the most plausible explanation for the presence of phylogenetic signals in the estimates of pollen temperature tolerance is the large‐scale biogeographic patterns. Specifically, closely related lineages often occur in spatially proximate regions, potentially leading them to inhabit more similar environments by chance, compared to more distantly related species (Lancaster and Humphreys 2020).

Yet, it is important to treat these findings with caution, as our data set includes a relatively small number of species (< 200 species out of 300K of total angiosperm diversity) and is strongly biased towards a few plant families, primarily representing species from temperate climates (e.g., Caryophyllaceae, Rosaceae). Thus, more research on PG and PTG is needed to assess pollen performance under different temperatures globally, particularly in wild plants occurring at lower latitudes (Rosbakh et al. 2018). This expanded research effort will shed more light on the evolution of pollen temperature tolerance.

### 4.5 Drivers of Interspecific Pollen Thermal Limits

The analysis of among‐species variation in pollen cardinal temperatures only partially supported our hypothesis that climate is the major driver of pollen thermal limits. Specifically, we found that pollen from species growing in colder climates, such as high elevations and latitudes, tended to exhibit greater cold tolerance compared to species inhabiting lower elevations and latitudes. Climates with low MATs (a proxy used in our analysis) are characterized by generally cool growing conditions and occasional nocturnal freezing (Geiger, Aron, and Todhunter 2009; Körner 2021). Consequently, plants in these environments likely evolved enhanced pollen cold tolerance to ensure successful fertilization (e.g., Rosbakh and Poschlod 2016; Wagner et al. 2016). The lack of variability in *Tmax* values along gradients of temperature favorability and variability further supports the theory that plant heat tolerance is physiologically constrained by high temperatures, as discussed above.

Contrary to our expectations and the ‘climate variability hypothesis’ (Cuesta et al. 2020; Lancaster and Humphreys 2020), we found that plants in more continental climates do not produce pollen with greater tolerance to both low and high temperatures, despite the large temperature ranges pollen may encounter at anthesis. One possible explanation is that plants in highly seasonal climates may time their reproductive phases to avoid periods of extreme temperatures, thereby reducing the selective pressure for broader pollen temperature tolerance. Additionally, the observed patterns in pollen temperature tolerance may reflect a geographic bias in our data set, as a substantial portion of the pollen cardinal temperature and distribution data originated from European regions strongly influenced by oceanic climates. This regional overrepresentation may skew the results by emphasizing species adapted to stable, cool environments while underrepresenting species from more variable or extreme climates. To gain a more comprehensive understanding of global patterns, future studies should incorporate data from a wider range of geographic regions and climatic conditions, ensuring representation from both stable and highly seasonal environments. In analyzing the influence of cultivation status and growth on pollen thermal limits, we only detected significant differences in pollen heat tolerance between herbaceous and woody species. Specifically, the pollen of herbaceous plants demonstrated enhanced performance at significantly higher temperatures, with *Tmax* values of 39.4°C, as opposed to 37.3°C in woody species. We attribute this pattern to the distinct thermal conditions experienced by woody and herbaceous flowers during anthesis, owing to their contrasting position on the stem at varying heights above the ground. The relatively slow air movement close to the ground, coupled with the efficient heat accumulation capacity of vegetation (Geiger, Aron, and Todhunter 2009; Körner 2021), often results in situations where pollen during presentation, dispersal and germination, experience temperatures considerably higher than ambient conditions (Dietrich and Körner 2014). This environmental selection pressure likely contributes to the observed superior heat tolerance in pollen of herbaceous plants. Differences in the lifespans of herbaceous and woody plants may also explain this pattern. Annual plants, which are typically herbaceous, have short and rapid life cycles, leading them to prioritize heat tolerance to ensure growth, flowering, and seed setting before adverse conditions arise.

## 5 Conclusions and Perspectives

In conclusion, our study of pollen temperature tolerance has revealed several crucial insights into the thermal limitations of pollen performance. First, we revealed that pollen performance is predominantly constrained to average temperatures ranging from 9.0°C to 42.4°C, with an optimal temperature for germination and PTG (*Topt*) at 26.5°C, with a few examples that can tolerate temperatures as low as −5°C and as high as 66.9°C. The correlation between low‐temperature tolerance in leaves and pollen suggests shared molecular adaptations, but the lack of a relationship for heat tolerance implies different mechanisms may be at play in these two tissues.

Second, the analysis of pollen thermal limits in 11 cultivated species revealed notable plasticity in intraspecific pollen temperature tolerance (particularly at the ‘cold’ end), most likely influenced by local growing conditions and/or breeding process. The lower variation in pollen heat tolerance across the study cultivars (populations) therefore raises serious concerns about the adaptability of plant sexual regeneration in the face of increasing high‐temperature stress from climate warming, although these findings alone cannot fully assess the adaptive potential of pollen thermal tolerance, as broader genetic assessments or selection experiments would be necessary.

Third, climate emerged as a significant driver of pollen thermal limits, with pollen of species in colder, stable climates displaying better cold tolerance. Further, we revealed considerable differences in temperature tolerance between woody and herbaceous plants that we attribute to unique thermal conditions during anthesis that pollen of the corresponding plants experience. Finally, phylogenetic analysis indicates conservation in pollen thermal limits at the family level, suggesting their shared evolutionary history.

These findings have two profound ecological implications. First, as pollen represents the most temperature‐sensitive stage in the plant reproductive cycle, the relatively low heat adaptability observed in this study raises concerns about the resilience of plant sexual reproduction to the temperature extremes associated with global climate change. In particular, the low heat tolerance in cultivated species underscores the potential challenges that modern agriculture may face as anthropogenic warming alters local climates. This highlights the need for screening pollen heat tolerance as an integral part of plant breeding programmes, with the goal of developing genotypes capable of withstanding high temperatures (Kakani et al. 2002, 2005).

Second, the close link between the pollen thermal limits and climate suggests that pollen temperature tolerance could serve as an alternative mechanism explaining species distribution patterns (Rosbakh and Poschlod 2016; Rosbakh et al. 2018). Specifically, the restriction of PG and PTG by temperature exceeding its physiological limits should ultimately result in poor seed production. Consequently, the reduction in reproductive output will, in turn, affect species' distribution, limiting their capacity to expand their geographical range or to maintain existing populations (Grubb 1977; Pigott and Huntley 1981; Turnbull, Crawley, and Rees 2000). Therefore, integrating knowledge of pollen thermal limits into future studies, a key precursor to seed production, will advance our understanding of species' distribution along climatic gradients and enhance our ability to predict the effects of anthropogenic climate change on plant geographic ranges.

While our study sheds new light on critical aspects of pollen temperature tolerance, it is imperative to acknowledge its limited scope. The most surprising finding of the study is that, despite its critical importance for plant science, the data on pollen thermal limits were available for only ca. 200 species only (i.e., 0.06% of the global vascular plant diversity). Hence, more work is necessary to study pollen thermal limits across the world's flora, especially in the non‐temperate regions. Additionally, future research should consider the precise temperature conditions pollen experiences during anthesis. The MAT and TAR used in this study as proxies for local temperature conditions are coarse and mask local‐scale variation in temperatures. Further studies are also needed to consider the thermotolerance of other pollen stages, such as dispersal and development in the anther, which might differ in their tolerance to low and high temperatures compared to germinating pollen and growing pollen tubes (Pacini and Dolferus 2019). Finally, to comprehensively understand pollen thermal limits, other potential drivers, such as flower morphology, flowering phenology and pollination type, should be considered in subsequent studies. Flower morphology, including the shape, size and structure of the flower, can influence how efficiently pollen is transferred and how it responds to temperature fluctuations. For instance, flowers with specialized structures like long corolla tubes may exhibit different thermal limits compared to more open, shallow flowers (Dietrich and Körner 2014). Furthermore, flowering phenology, or the timing of flower blooming, closely interacts with temperature dynamics (Wagner et al. 2016). Flowers blooming during colder months, like between March and May in temperate climates, would necessitate pollen adaptation to lower temperatures as compared to their counterparts blooming in warmer months (Rosbakh and Poschlod 2016). Therefore, late‐flowering species might exhibit higher heat tolerance, while early‐flowering species may display higher cold tolerance. In regard to pollination type, whether wind, insects or other vectors are involved, it also affects pollen thermal limits, reflecting diverse ecological strategies. For example, anthers of insect‐pollinated plants are often located deep inside the flower, exposing them to a warmer thermal environment than those of wind‐pollinated plants, which typically have anthers outside the flower and are thus exposed to lower temperatures (Whitehead 1983; Welsford, Midgley, and Johnson 2014). Consequently, it is expected that the cardinal temperatures of PG in insect‐pollinated species would be higher than those in wind‐pollinated species.

## Conflicts of Interest

The authors declare no conflicts of interest.

## Supporting information

Supporting information.

## Data Availability

The original contributions presented in the study are included in the article/Supplementary Material, further inquiries can be directed to the corresponding author/s. Data (pollen cardinal temperatures and species distribution) and R code used in the analysis are available at Zenodo: https://zenodo.org/records/13843132.
