# Supplementary material for: Patterns and Drivers of Pollen Temperature Tolerance
Source: Plant Cell Environ. 2024 Oct 24;48(2):1366–79. doi: 10.1111/pce.15207 (PMC11695751; doi:10.1111/pce.15207)
Supplement: Supplementary file 1 — Supporting information. [file PCE-48-1366-s001.docx]

**Supplementary materials**

Appendix 1

Matrix 1 – Intraspecific values

**
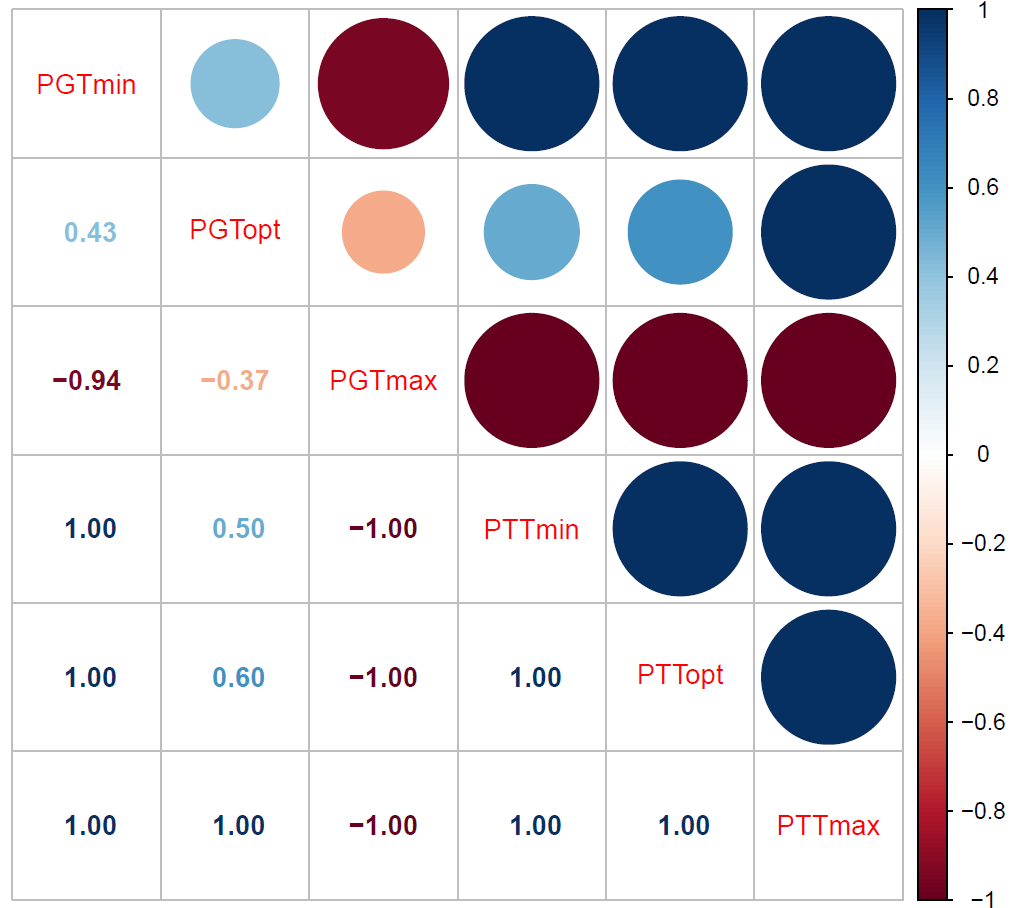
**

Matrix 2 – Interspecific values


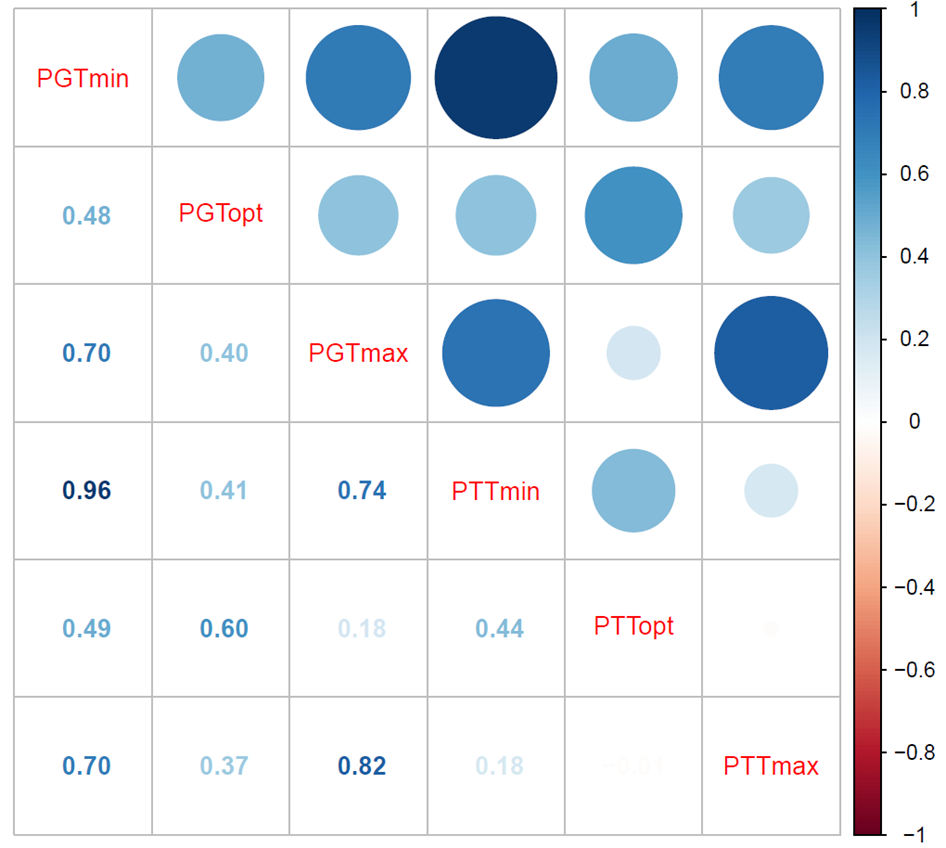


Figure A1. Correlation matrices (Spearman rank correlation coefficient) for cardinal temperatures of pollen germination (PG) and pollen tube growth (PTG) at intraspecific (first matrix) and interspecific (second matrix) levels. Significant results (p<0.05) are filled cells. The colour indicates the nature of the relationship (blue – positive, red – negative); the circle size indicates the strength of the correlation (the larger the stronger). Tmin, Topt, Tmax are minimum (base), optimal and maximum (celling) temperatures of pollen performance, respectively.

******

Figure A2. Distribution of minimal (the inner ring), optimal (the middle ring) and maximal (the outer ring) temperatures of pollen temperature tolerances across the species phylogeny. Grey cells are species with unavailable data.

Appendix 2

Table A1. Intraspecific trait variation in pollen temperature limits in eleven cultivated plant species. Tmin, Topt and Tmax are minimal, optimal and maximal pollen tolerance temperatures; CV – coefficient of variation.

| **Species** | **Characteristic** | **n** | ***Tmin*** | | | | ***Topt*** | | ***Tmax*** | |
| --- | --- | --- | --- | --- | --- | --- | --- | --- | --- | --- |
|  |  |  | Mean | | CV | | Mean | CV | Mean | CV |
| *Arachis hypogaea* (Groundnut) | A legume cultivated in (sub)tropical climates | 44 | 14.34 | | 11.79 | | 32.20 | 10.25 | 43.24 | 5.74 |
| *Brassica napus* (Rapeseed or canola) | An oil seed crop mainly grown in temperate climates | 52 | 6.46 | | 31.09 | | 24.27 | 15.90 | 33.58 | 5.67 |
| *Capsicum annuum* (Bell or chili pepper) | A vegetable typically grown in warm and temperate regions | 36 | 12.36 | | 10.23 | | 27.58 | 10.19 | 41.07 | 1.74 |
| *Cocos nucifera* (Coconut) | A fruit commonly cultivated in tropical regions | 44 | 13.78 | | 17.38 | | 27.40 | 5.74 | 40.51 | 8.04 |
| *Glycine max* (Soybean) | A legume grown in diverse climates | 184 | 11.32 | | 22.33 | | 31.29 | 11.85 | 47.12 | 3.98 |
| *Gossypium hirsutum* (Upland cotton) | A fiber crop primarily grown in (sub)tropical climates | 52 | 12.62 | | 14.32 | | 28.61 | 7.67 | 43.23 | 3.49 |
| *Juglans regia* (Walnut) | A nut that thrives best in temperate regions | 20 | 13.65 | | 13.71 | | 30.00 | 12.09 | 40.37 | 3.71 |
| *Pennisetum glaucum* (Pearl millet) | A cereal well-adapted to hot and dry climates | 56 | 9.38 | | 24.26 | | 28.28 | 6.93 | 47.35 | 4.58 |
| *Pistacia vera* (Pistachio) | A nut primarily cultivated in mediterranean climate | 26 | 6.70 | | 37.65 | | 25.46 | 10.84 | 40.79 | 4.11 |
| *Saintpaulia ionantha* (African violet) | An ornamental plant cultivated in tropical climates | 60 | 4.62 | | 72.81 | | 25.41 | 5.21 | 46.54 | 11.91 |
| *Sorghum bicolor* (Sorghum) | A cereal cultivated in (sub)tropical regions | 38 | 11.37 | | 47.08 | | 29.75 | 12.77 | 42.55 | 4.45 |
|  | **Mean** | | | 10.60 | | 27.51 | 28.20 | 9.95 | 42.40 | 5.22 |
